# Non-communicable disease care delivered by emergency medical teams during the Gaza Health Response in 2024–2025: an observational analysis of WHO Minimum Data Set Reports

**DOI:** 10.1007/s11739-026-04483-y

**Published:** 2026-08-07

**Authors:** Luca Pigozzi, Husam Abuolwan, Heba Al Najjar, Shannon Barkley, Souheil Reaiche, Nabil Tabbal, Onur Mavi, Tatsuhiko Kubo, Ana Fernandez-Somoano, Flavio Salio, Rafael Castro-Delgado

**Affiliations:** 1 World Health Organization (WHO), Occupied Palestinian Territory Country Office, East Jerusalem/Gaza, Occupied Palestinian Territory; 2 Department of Medicine, Faculty of Medicine and Health Sciences, University of Oviedo , Julian Claveria, 6, 33006 Oviedo, Spain; 3 Research Group On Prehospital Care and Disasters, GIAPREDE, Health Research Institute of the Principality of Asturias, Oviedo, Spain; 4 World Health Organization (WHO), Eastern Mediterranean Regional Office, Cairo, Egypt; 5 World Health Organization (WHO), Emergency Medical Teams, Geneva, Switzerland; 6 Humanitarian and Disaster Management Department, World Health Organization (WHO), Geneva, Switzerland; 7 Department of Public Health and Health Policy, Graduate School of Biomedical and Health Sciences, Hiroshima University, Hiroshima, Japan; 8 Department of Multi-Sectoral Preparedness and Strategic Coordination, Graduate School of Medicine, Kyoto University, Centre for Health Security, Kyoto, Japan; 9 Oviedo University, Instituto Universitario de Oncología del Principado de Asturias (IUOPA), Oviedo, Spain; 10 Spanish Consortium for Research On Epidemiology and Public Health (CIBERESP), Instituto de Salud Carlos III, Madrid, Spain; 11 Institute of Health Research of the Principality of Asturias-Foundation for Biosanitary Research of Asturias (ISPA-FINBA), Oviedo, Spain; 12 World Health Organization (WHO), Global Health Emergency Corps, Geneva, Switzerland

**Keywords:** Emergency medical team, Non-communicable disease, Primary healthcare, Humanitarian response, Gaza

## Abstract

The objective is to describe patterns of noncommunicable disease (NCD) service delivery and the operational roles of the different Emergency Medical Team (EMT) types—Type1 mobile (outpatient mobile primary care), Type1 fixed (outpatient static primary care), and Type2 (inpatient and surgical care)—in NCD care during the emergency response in Gaza throughout 2024–2025. This study analyzed routinely collected aggregate data from the World Health Organization EMT Minimum Data Set reported by EMTs across Gaza between January 2024 and December 2025. A total of 317,624 NCD consultations were included. Consultations for hypertension, diabetes, asthma/chronic obstructive pulmonary disease, cardiovascular disease, and epilepsy were examined across the different EMT types. Temporal trends were analyzed across six predefined conflict phases using non-parametric statistical testing. Statistically significant differences were observed for NCD consultations across EMT types, suggesting complementary roles. Type1 mobile teams showed the highest proportions of new diagnoses, indicating a frontline case-detection role. Type1 fixed teams demonstrated intermediate follow-up new diagnosis ratios, functioning primarily as chronic disease management hubs. Type2 teams managed the highest burden of acute exacerbations (87.6%), reflecting their role in severe complication management. NCD consultation patterns varied across conflict phases, with increases during ceasefire periods. Aggregate data from EMTs in Gaza revealed patterns consistent with comprehensive NCD care, including case detection, longitudinal clinical management, and acute exacerbation care across EMT types. These findings highlight the importance of integrating NCD services early in humanitarian responses, sustaining continuity of care, and reducing downstream pressure on higher-level services during conflicts.

## Introduction

Emergency medical teams (EMTs) are organized groups of health professionals deployed to provide rapid, quality-assured clinical care during disasters and public health emergencies, globally led by the World Health Organization (WHO) [1]. EMTs represent a critical component of emergency health responses by deploying surge capacity, restoring essential services, and supporting health systems when local resources are insufficient or nonfunctional. Their adherence to minimum technical and operational standards ensures a predictable, scalable, and coordinated response, particularly in settings where national health systems are overwhelmed [2].

Since October 2023, communities have been exposed to intense warfare resulting in loss of lives, injuries, and mass population displacement, as well as severe destruction and damage to civilian infrastructure, including healthcare facilities [3, 4], with more than 50% being partially or completely nonfunctional [5]. Adequate access to primary, secondary, and specialized healthcare services is severely hampered by security constraints. The lack of adequate healthcare workforce, pharmaceutical products, medical consumables, electricity, and communication infrastructure further compromises the safe delivery of adequate health services across Gaza.

The pre-conflict burden of NCDs in Gaza was substantial. Hypertension affected an estimated 25% of adults and diabetes approximately 12%, in alignment with regional trends [6]. Cardiovascular disease, asthma/chronic obstructive pulmonary disease (COPD), and epilepsy represented additional significant sources of morbidity. Before October 2023, NCD care in Gaza was primarily delivered through a network of facilities managed by the Ministry of Health, the United Nations Relief and Works Agency for Palestine Refugees in Near East, and several non-governmental organizations. However, pre-existing challenges including chronic medication shortages and limited diagnostic capacity had already constrained the quality and continuity of NCD management for years before the onset of the current emergency [4, 6]. During the ongoing health emergency, disruptions in continuity of care, combined with displacement and deteriorating living conditions, have notably increased the risk of complications, disease exacerbations, and preventable mortality among patients with chronic diseases [7–12].

Within this framework, EMT Type1 (mobile and fixed), designed to deliver outpatient and comprehensive primary care services, and EMT Type2, intended to provide also inpatient and surgical capacity, have played a pivotal role in addressing healthcare gaps during the acute phases of the Gaza health response between 2024 and 2025 [13]. By providing accessible frontline services, EMT Type1 and Type2 support the management of stable NCD patients, ensure the availability of essential medications, facilitate continuity of care, stabilize and appropriately refer patients presenting with acute exacerbations and complications [14, 15].

This study aims to comprehensively analyze and characterize the role of different EMT types in sustaining and delivering essential NCD care services during the Gaza health response throughout 2024–2025.

## Methods

### Study design and data source

This study analyzed routinely collected patient data reported by national and international EMTs during the Gaza health response across 2024 and 2025. Data were collected using the EMT Minimum Data Set (MDS), a standardized tool for disaster-related health data collection (with adaptation for conflict settings) endorsed by the WHO since 2017 [16]. The study period spanned 24 months, from 1 January 2024 to 31 December 2025.

The following variables were extracted from the EMT MDS: date of reporting, facility name, EMT type, and cumulative case count for five health events, notably hypertension (HTN), diabetes (DM), asthma/COPD, cardiovascular diseases (CVD) and epilepsy. Total NCD consultations per report were computed as the aggregate sum of all five categories, including both new diagnosis and follow-up cases. These five conditions correspond to the NCD categories defined within the MDS, representing leading causes of NCD-related morbidity and mortality in humanitarian settings and are assessable with basic clinical capacity available at all EMT types [1, 16]. MDS reports containing zero NCD cases and collected by EMT Specialized Care Teams (SCTs) were excluded. Temporal analysis was conducted at monthly resolution to enable comparison with conflict-related events. All figures are presented as aggregated totals across all age groups.

### Participants

A total of 6873 daily or weekly MDS reports, representing 317,624 NCD consultations were included in the analysis. Reports were submitted by 21 EMTs to the WHO-led EMT Coordination Cell (EMTCC). Participating teams comprised 7 Type1 mobile, 9 Type1 fixed, and 5 Type2 teams. These EMTs were deployed across all five governorates of Gaza at different stages of the health response throughout 2024 and 2025 [13, 17].

### Conflict-phases definition

Six conflict phases were defined a priori based on documented key events [17]. Phase boundaries were established prior to analysis and were not adjusted post hoc (Table 1).

**Table 1:** Conflict phases, periods and defining key events

Phase	Period	Key events
Escalation I	January–May 2024	Ongoing operations in Gaza City, North Gaza, and Khan Younis, driving mass displacement to Rafah; Rafah crossing remained open
Escalation II	June–December 2024	Large-scale operations following the Rafah offensive, causing further displacement toward Khan Younis and the Middle Area; Rafah crossing closed
Ceasefire I	January–February 2025	Ceasefire agreement implemented on 19 January 2025
Resumption	March–April 2025	Collapse of negotiations; hostilities resumed on 18 March 2025
Escalation III	May–September 2025	Renewed major offensive operations across the Gaza Strip
Ceasefire II	October–December 2025	Ceasefire agreement in effect from 10 October 2025

### Data gathering

The Gaza health response unfolded in a context of rapid and large-scale EMT deployment. The EMTCC was established in Gaza and Cairo under the leadership of WHO, with the technical support of experts from the European Union Civil Protection Mechanism and the Japan International Cooperation Agency (JICA). Following endorsement by the local health authorities, the MDS began to be implemented by deployed EMTs from January 2024 onward. Between January 2024 and December 2025, a total of 57 international and 2 national EMTs deployed across Gaza contributed to this system.

The MDS tool used in Gaza included 92 variables across four sections: Team Information, Daily Summary, Health Events and Procedures, and Needs and Risks. Teams were requested to submit daily or weekly reports, providing information critical to support coordination of the health response by local authorities and WHO.

Reports were compiled using the “MDS Maker” tool developed by JICA, generating CSV files submitted via email or WhatsApp. Where internet access was unavailable, reports were delivered in person when security conditions allowed. Upon receipt, WHO and JICA teams screened the data for duplicates and inconsistencies before storing the cleaned dataset on the “MDS Feedback Maker + ” secure cloud platform. Selected outputs were made available on a public dashboard [13]. All data within the MDS are aggregated and fully anonymized at the point of collection.

### Statistical analysis

All statistical analyses were performed using Python 3.12 (Python Software Foundation). Data management and aggregation were performed with pandas 2.2; numerical computation with NumPy 1.26; non-parametric tests (Kruskal–Wallis, Mann–Whitney U, and Jonckheere–Terpstra) and ordinary least squares (OLS) regression with SciPy 1.13 and statsmodels 0.14, respectively; and data visualization with matplotlib 3.10 and seaborn 0.13. Reporting follows the STROBE guidelines for observational studies [18]. Generative AI (Claude Sonnet 4.6) was used to assist in drafting portions of the Python code for data processing and statistical analysis. All AI-generated code was subsequently reviewed, manually tested, and validated by a software developer to ensure accuracy, logic, and reproducibility. The authors remain fully accountable for the integrity of the final code and the resulting data.

### Ethical considerations

This study analyzed aggregate and facility-level data only. No individual patient records, identifiers, or clinical notes were accessed at any stage of the analysis. The dataset was collected under the WHO MDS operational framework as part of routine EMT coordination activities. The study was conducted in accordance with the Declaration of Helsinki and was approved by the Ethical Committee for Responsible Research and Innovation of University of Oviedo (ref. number 69/2026).

## Results

### Distribution of NCD consultations across EMT types

**Fig. 1:**
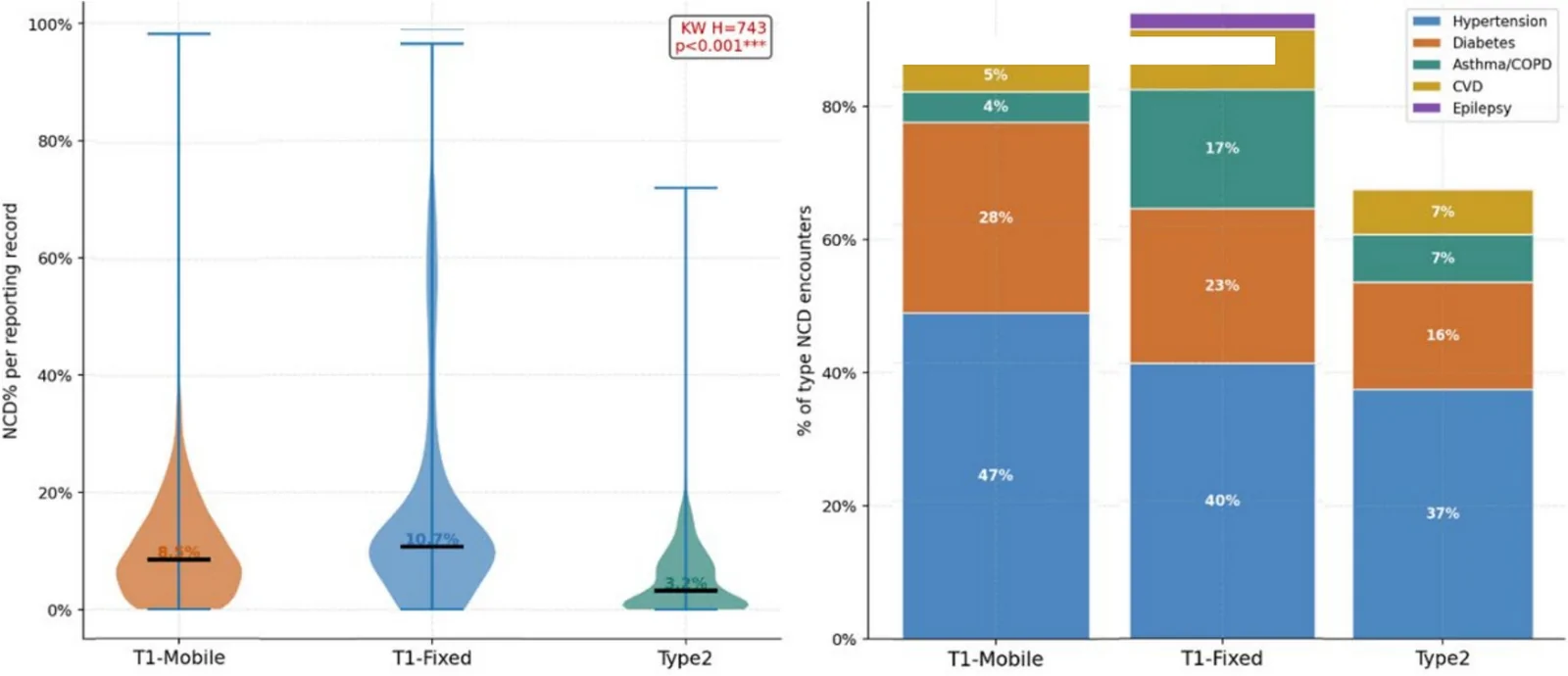
Left: distribution of NCD% per reporting record by EMT type (violin plots; all pairwise *p* < 0.001). Right: NCD condition composition by EMT type (% of type NCD total)

Across 6873 reported MDS records, a total of 2,953,150 all-cause consultations were documented, of which 317,624 were accounted as NCD cases, representing 10.8% of total consultations (Table 2). The remaining 89.2% of consultations comprised a broad range of other health events, including traumatic injuries, maternal and child health conditions, acute infectious diseases, and other undifferentiated acute presentations, consistent with the comprehensive clinical scope of deployed EMTs. Marked and statistically significant differences in the proportion of NCD consultations (NCD%) were observed across EMT types (Table 2; Fig. 1 left). Type2 teams showed the lowest NCD proportion (7.5%) compared to Type1 fixed (11.9%) and Type1 mobile teams (11.9%). Although statistically significant at the record level (Mann–Whitney, *p* < 0.001), the difference between Type1 mobile and Type1 fixed teams was clinically negligible, with aggregate NCD proportions of 11.9% in both types. Despite this lower proportional contribution, Type2 teams accounted for the majority of exacerbated NCD cases (87.6%). Analysis of exacerbation proportions revealed a clear gradient in case severity across EMT types, with statistically significant differences (*p* < 0.001), being 61.8% for Type1 fixed teams and 36.7% for Type1 mobile teams (Table 2).

**Table 2:** NCD consultations across three EMT types—Gaza 2024–2025

EMT Type	MDS records	All consultations	NCD consultations	NCD %	Exacerbation %
Type1 mobile	2830	845,950	100,766	11.9%	36.7%
Type1 fixed	2553	1,340,719	159,050	11.9%	61.8%
Type2	1490	766,481	57,808	7.5%	87.6%
Total	6873	2,953,150	317,624	10.8%	58.4%

The distribution of NCD% at the reporting-record level further illustrates these differences (Fig. 1, left). Type1 mobile and fixed teams showing similar central tendencies and dispersion, whereas Type2 teams demonstrate a distinctly lower distribution. Additionally, variation in the composition of NCD conditions by EMT type is presented in Fig. 1 (right), highlighting differences in case mix across service delivery levels and clinical capacity.

### New diagnosis and follow-up rates across EMT types

New diagnosis proportions varied markedly across EMT types, with Type1 mobile teams demonstrating the highest proportion of newly identified cases across all NCD conditions. For HTN, 33.0% of consultations managed by Type1 mobile teams were new diagnoses, compared to 8.6% in Type1 fixed teams and 2.2% in Type2 teams (H = 1139.8; *p* < 0.001). A similar pattern was observed for DM, with new diagnosis proportions of 29.1% in Type1 mobile, 7.6% in Type1 fixed, and 1.7% in Type2 teams. The Jonckheere–Terpstra test confirmed a significant descending gradient in new diagnosis proportions across all five conditions (Type1 mobile → Type1 fixed → Type2; HTN: z =  − 22.03; DM: z =  − 22.79; Asthma/COPD: z =  − 19.10; CVD: z =  − 11.19; Epilepsy: z =  − 8.28; all *p* < 0.001), consistent with the hypothesized gradient of decreasing case-detection activity from Type1 mobile to Type1 fixed to Type2 teams.

**Fig. 2:**
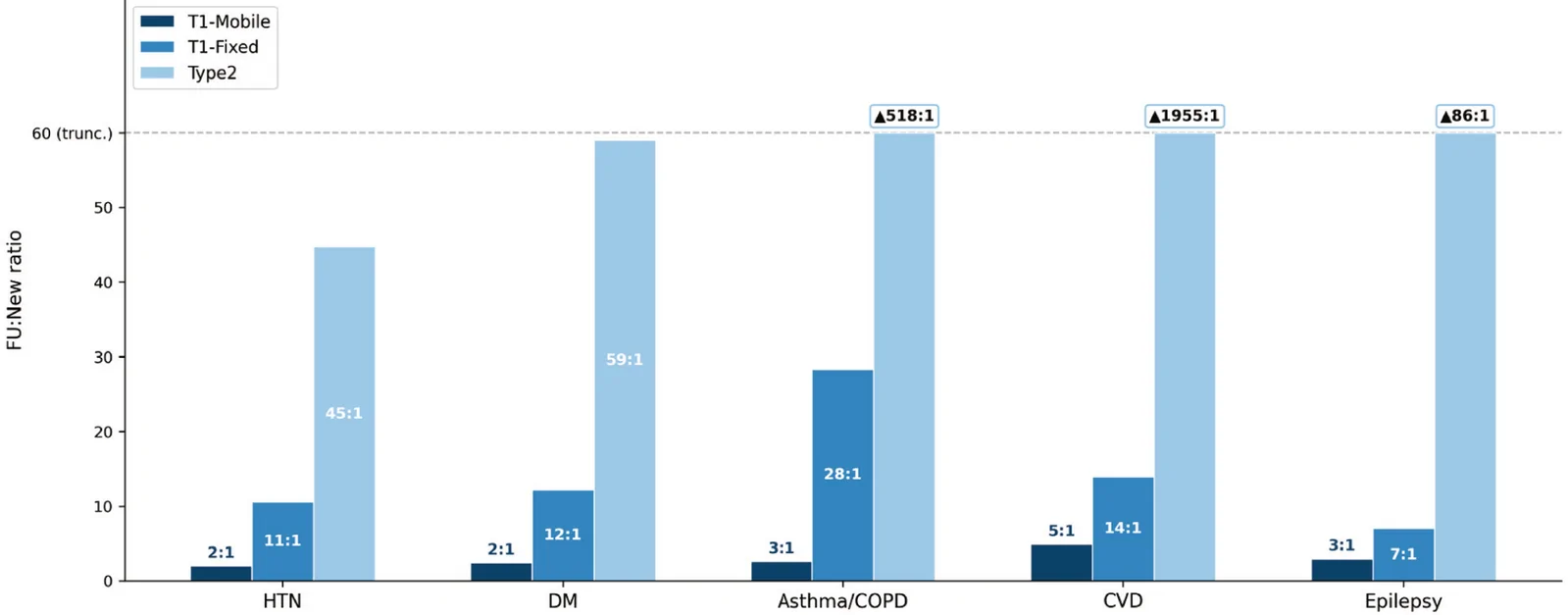
Follow-up: to New Diagnosis ratios by NCD condition and EMT type (bars capped at 60 for readability; actual values shown as labels)

Follow-up to new (FU:New) ratios further differentiated the operational profiles of EMT types (Table 3; Fig. 2). Across all NCD conditions, Type1 mobile teams showed the lowest ratios, indicating a comparative greater contribution to new case identification. In contrast, Type1 fixed teams demonstrated intermediate ratios, while Type2 teams demonstrated substantially elevated ratios, reflecting a predominance of follow-up care.

**Table 3:** Follow-up to New Diagnosis ratios by condition and EMT type—Gaza 2024–2025

NCD condition	Type1 mobile		Type1 fixed		Type2	
	New %	FU:New	New %	FU:New	New %	FU:New
HTN	33.0%	2.0:1	8.6%	10.6:1	2.2%	44.8:1
DM	29.1%	2.4:1	7.6%	12.2:1	1.7%	59.0:1
Asthma/COPD	27.9%	2.6:1	3.4%	28.3:1	0.2%	518:1
CVD	16.9%	4.9:1	6.7%	13.9:1	0.05%	1,955:1
Epilepsy	25.2%	3.0:1	12.5%	7.0:1	1.1%	86:1

*HTN* hypertension, *DM* diabetes mellitus, *CVD* cardiovascular disease, *FU* follow-up, *New* new diagnosis

Condition-level breakdowns are available only for the five MDS tracer conditions

NCD consultations not disaggregated by condition are excluded from this table

For CVD, the FU:New ratio increased from 4.9:1 in Type1 mobile teams to 13.9:1 in Type1 fixed teams and reached 1955:1 in Type2 teams. Similarly, asthma / COPD showed ratios of 2.6:1 in Type1 mobile, 28.3:1 in Type1 fixed, and 518:1 in Type2 teams. HTN followed the same gradient, with FU:New ratios of 2.0:1 (Type1 mobile), 10.6:1 (Type1 fixed), and 44.8:1 (Type2). Across additional NCD conditions, including DM and epilepsy, this pattern was consistently observed, with progressively higher FU:New ratios from mobile to fixed to Type2 teams, suggesting a continuum of care of NCD across EMT levels (Table 3).

**Fig. 3:**
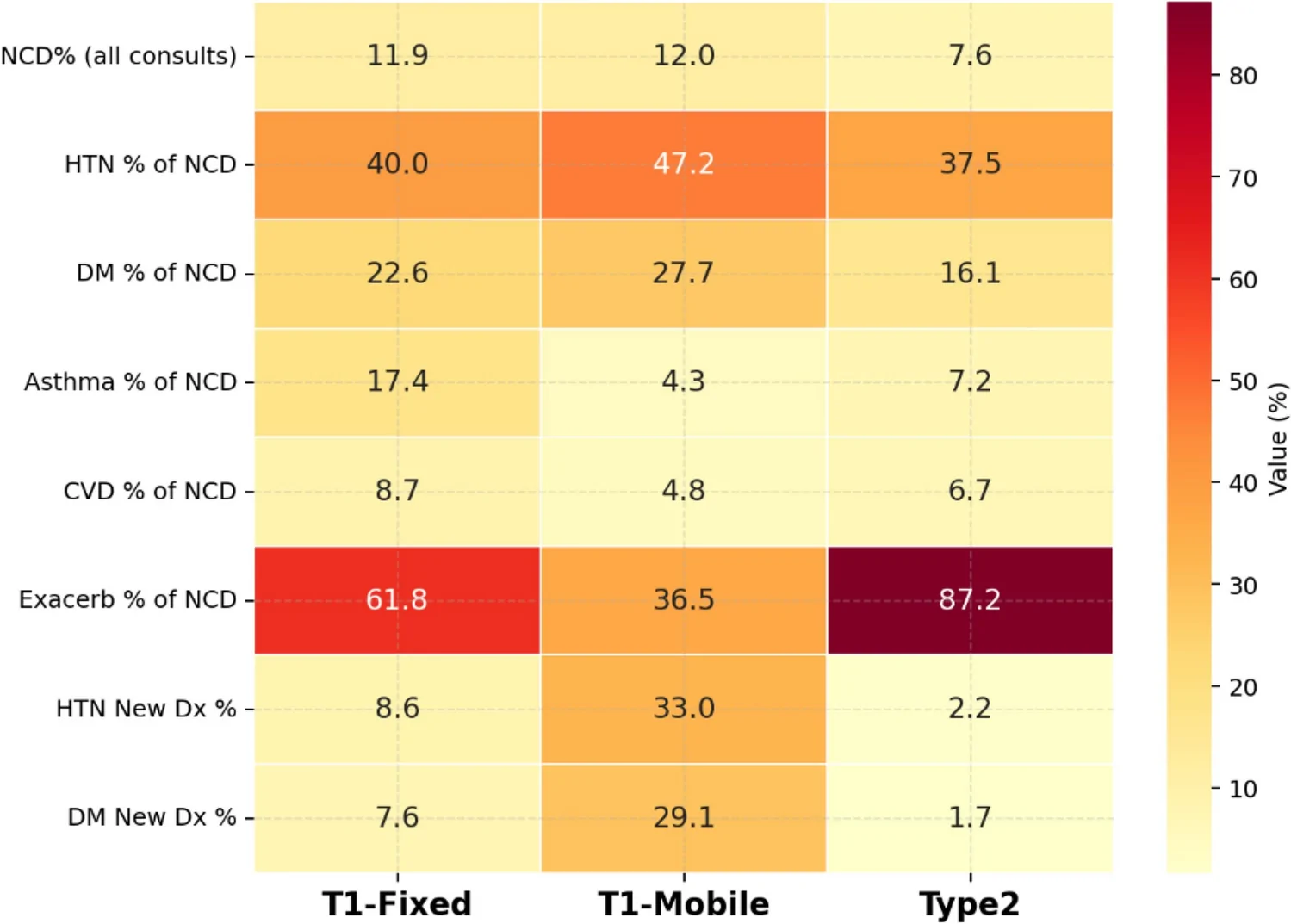
Heatmap of NCD performance indicators across three EMT types

The distribution of FU:New ratios across EMT types is illustrated in Fig. 2. For visualization purposes, extreme FU:New values observed in Type2 teams were truncated, with actual values indicated. Differences in NCD-related performance indicators across EMT types are further summarized in Fig. 3, which presents a heatmap of key metrics, including NCD%, new diagnosis rates, follow-up ratios, and exacerbation burden. Distinct patterns were observed across the three EMT types, reflecting variation in service delivery characteristics.

### NCD distributions by conflict phases and EMT types

**Fig. 4:**
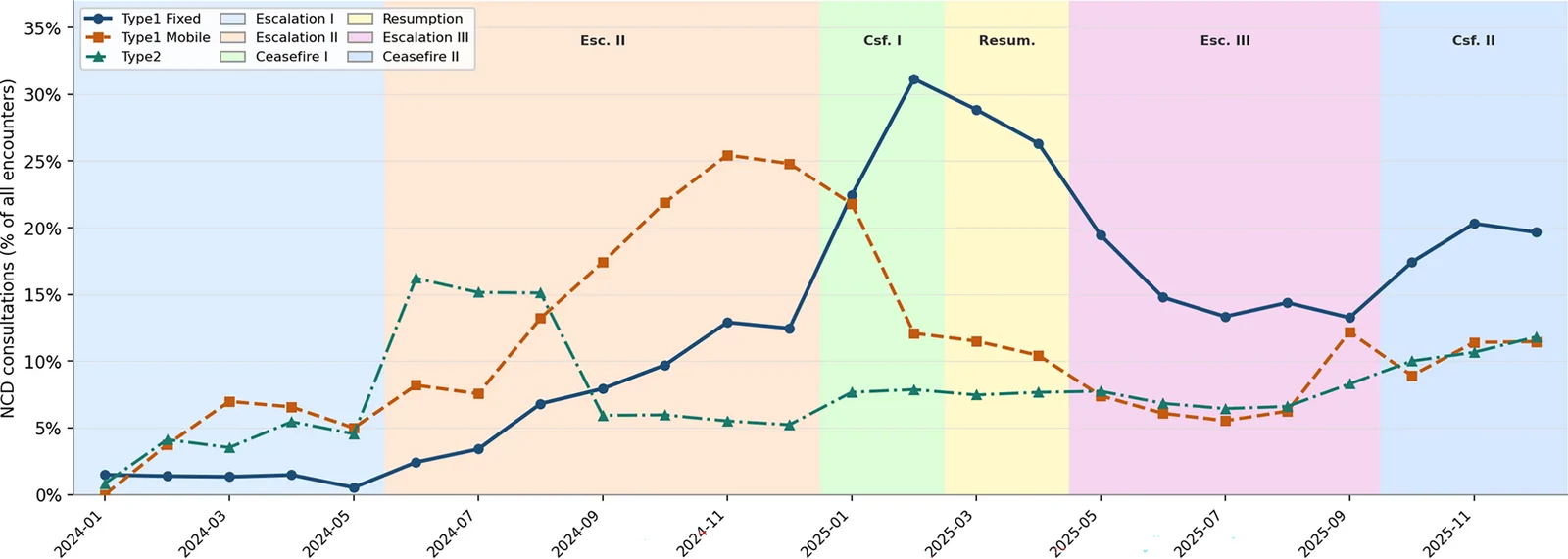
Monthly NCD % by EMT type across conflict phases in 2024–2025

Monthly trends in NCD% across conflict phases are presented in Fig. 4. Statistically significant variations in NCD% were observed across conflict phases for all three EMT types. Type1 fixed teams showed the strongest phase-level variation (p < 0.001), with NCD% increasing progressively from 1.2% during “Escalation I” to a peak of 27.6% during “Resumption” phase before remaining elevated at 19.1% during “Ceasefire II”. Type1 mobile teams also showed statistically significant, although less pronounced, variation across phases (*p* = 0.009), averaging 16.9% during “Ceasefire I” with a peak in January of 21.7% and declining to 7.5% during “Escalation III”. Type2 teams showed the most modest degree of variation (*p* = 0.024), with NCD% remaining consistently below 11% throughout all conflict phases.

Ordinary least squares regression confirmed a statistically significant upward trend in NCD% over the 24-month study period for Type1 fixed teams (β =  + 0.956 percentage points/month; *p* < 0.001), consistent with the progressive expansion of NCD services and increasing chronic disease caseload as the conflict became protracted. Type1 mobile and Type2 teams did not show a statistically significant linear trend over time (*p* > 0.05).

Exacerbation rates also varied statistically significantly across conflict phases (*p* = 0.012). The lowest median exacerbation proportion was recorded during “Escalation I” (5.7%), while the highest was observed during the “Resumption” phase (79.7%). Notably, exacerbation proportions across all EMT types tended to be higher during ceasefire phases than during active escalations. The Jonckheere–Terpstra test confirmed a significant ascending trend in exacerbation proportion across EMT types (z = 12.97; *p* < 0.001), consistent with the hypothesized gradient of increasing acute complication burden from mobile to Type2 teams.

Both ceasefire periods were associated with marked shifts in NCD consultation patterns, although the magnitude and direction of these changes differed by EMT types. During “Ceasefire I” the overall NCD% across all types increased from 13.6% to 19.3%, driven primarily by a sharp increase in Type1 fixed teams (12.7% to 26.8%, representing an absolute increase of 14.1 percentage points (pp) and a relative increase of + 111%). In contrast, Type1 mobile demonstrated a decline during the same period (25.1% to 16.9%; − 8.2 pp; − 33%), while Type2 showed only a modest increase (5.4% to 7.8%; + 2.5 pp). During “Ceasefire II” NCD% increased across all EMT types. Type1 fixed team showed the largest absolute rise (13.9% to 19.1%; + 5.3 pp; + 38%), followed by Type2 (7.4% to 10.5%; + 3.1 pp; + 41%) and Type1 mobile (9.2% to 10.6%; + 1.4 pp; + 15%).

## Discussion

This study provides the first large-scale, facility-level analysis of NCD care delivered by EMTs during the Gaza health response across 2024–2025, suggesting a differentiation of roles across EMT types. The findings may indicate patterns consistent with the emergence of an operational continuum of NCD care, spanning case detection, clinical case management, and acute exacerbation care across EMT types.

Type1 mobile and fixed teams played a critical role during the early phases of the health response by ensuring continuity of care for patients with chronic conditions. Although the immediate urgency of NCD care is often less visible than trauma-related needs, interruptions in routine treatment rapidly lead to increased exacerbations among individuals living with NCDs [8]. This temporal lag is particularly relevant in complex emergencies, where the effects of interrupted care may not become immediately apparent but instead emerge over weeks to months, as patients present with acute complications that further increase hospital admissions, bed occupancy and pressure on limited services [19]. These findings reinforce the importance of integrating NCD services from the outset of emergency responses [10].

This study hints at a three-tier functional model of NCD care within EMT operations in Gaza. Type1 mobile teams primarily functioned as frontline access and case-finding level, with high proportions of new diagnoses, particularly for common health conditions such as HTN and DM. This may reflect both substantial unmet baseline healthcare needs and the impact of displacement, deteriorating living conditions, and reduced access to primary care services, though the possibility that some consultations classified as new diagnoses represent previously diagnosed patients re-presenting to a different EMT facility cannot be excluded given the absence of patient-level tracking data [20]. Simultaneously, the absence of specialized staff and structured screening programs at Type1 level suggests that many diagnoses were often opportunistic, relying on general clinical assessment rather than systematic screening [21]. In contrast, Type1 fixed teams predominantly functioned as chronic disease management hubs, characterized by substantially higher FU:New ratio and greater continuity of longitudinal care. Type2 teams, meanwhile, primarily managed acute exacerbations and severe complications, with exacerbation proportions exceeding 80% and minimal contribution to new diagnoses.

These observations might have important operational implications. Continuity of chronic disease care at the Type1 level is a critical determinant of downstream burden on higher-level services. Interruptions in access to essential medications and follow-up consultations are likely to contribute to increased acute presentations at Type2 facilities, which are considerably more resource intensive. This underscores the need to bolster NCD care into the essential service delivery package of Type1 and Type2 capacities. Type1 teams should be equipped with essential chronic medications, basic diagnostic tools, and simplified clinical protocols to sustain continuity of care, while Type2 teams should incorporate acute NCD exacerbation management as an anticipated operational function [10].

The analysis of NCD trends across conflict phases may further indicate a complex and phase-dependent dynamic with important implications for humanitarian NCD care planning. During the early escalation phase, NCD% remained relatively low across all EMT types, likely reflecting the broader orientation of the health response towards other interventions, such as emergency care and maternal and child health [8, 9, 22]. The progressive rise in NCD% during the subsequent phases, particularly in Type1 mobile teams, illustrates the growing recognition of chronic disease burden as the conflict evolved over time.

The ceasefire periods were associated with a particularly marked increase in NCD%, mainly in Type1 fixed teams. This pattern may be indicative of previously inaccessible patients with chronic conditions re-engaging with healthcare services when insecurity and movement restrictions temporarily decreased. However, this increase in healthcare utilization and NCD patients was not accompanied by a proportional reduction in exacerbation rates, suggesting that many patients sought care only after prolonged periods of treatment interruption and clinical deterioration, as previously documented in other conflict-affected settings [23, 24].

## Limitations

This study has several limitations inherent to the use of MDS aggregated data. Firstly, the absence of patient-level identifiers prevents longitudinal tracking across facilities, introducing the possibility of double counting, particularly among displaced populations. Consequently, some consultations categorized as new diagnosis may have represented previously diagnosed patients accessing different EMT services. This limitation is particularly relevant for EMT Type1 mobile services and may lead to an overestimation of their case-finding capacity if repeated presentations are misclassified as new diagnoses. However, the consistent patterns observed between EMT Type1 fixed and Type 2 services support the overall robustness of the findings.

Second, variability in reporting practices across EMTs, combined with limited diagnostic capacity, particularly at Type1 level, may have influenced case classification and contributed to underdiagnosis or misclassification of NCD conditions.

Furthermore, the MDS does not capture individual patient demographic information, such as age and sex, data on comorbidities, clinical outcomes, including mortality, treatment adherence, medication availability, and referral completion. Therefore, this study does not directly assess the impact of NCD care and the effectiveness of the continuity of care.

Finally, observed differences between EMT types may also reflect variation in deployment timing, geographic location, catchment population characteristics, and duration of deployment. These potential confounders were not systematically controlled for and should be considered when interpreting the findings.

## Conclusion

This study provides the first large-scale analysis of NCD care delivered by EMTs in a protracted conflict setting, based on over 317,000 NCD consultations conducted in Gaza between 2024 and 2025. The findings suggest an operational continuum of care across EMT types, ranging from frontline access and case-finding at the Type1 mobile level, to longitudinal chronic disease management within the Type1 fixed teams, and acute exacerbation care at Type2 tier. Phase-specific analysis further showed that ceasefire periods were associated with substantial surges in NCD demand, particularly at Type1 fixed facilities, likely driven by patients re-accessing healthcare services after extended interruptions in treatment and follow-up.

The persistently high burden of acute decompensation observed at the Type2 level, across all conflict phases, including ceasefire periods, highlights the clinical consequences of disrupted continuity of care and underscores the importance of early integration of NCD services within emergency responses. Strengthening Type1 capacity is, therefore, critical to preventing avoidable complications and reducing pressure on higher-level referral services.

Overall, these findings reinforce the operational principles outlined in EMT minimum standards [1] highlighting that effective NCD management in disasters and complex emergencies requires a coordinated, multi-level approach in which continuity of care is prioritized and each EMT type is appropriately prepared and equipped to fulfill complementary roles within the broader health response system.

## Data Availability

For operational sensitivity in an active conflict setting, only aggregated data may be available by the corresponding author upon reasonable request and with WHO EMTCC permission.

## Declarations

### Disclaimer

The opinions expressed in this article are those of the authors and do not reflect the official position of the World Health Organization (WHO). WHO takes no responsibility for the information provided or the views expressed in this article.
